# Suppression of airway allergic eosinophilia by *Hp*-TGM, a helminth mimic of TGF-β

**DOI:** 10.1111/imm.13528

**Published:** 2022-07-12

**Authors:** Caroline Chauché, Orhan Rasid, Anne-Marie Donachie, Caitlin M. McManus, Stephan Löser, Tiffany Campion, Josh Richards, Danielle J. Smyth, Henry J. McSorley, Rick M. Maizels

**Affiliations:** 1 Wellcome Centre for Integrative Parasitology, Institute of Infection, Immunity and Inflammation, University of Glasgow, Glasgow, UK; 2 Centre for Inflammation Research, University of Edinburgh, Queen’s Medical Research Institute, Edinburgh, UK; 3 Division of Cell Signalling and Immunology, School of Life Sciences, Wellcome Trust Building, University of Dundee, Dundee, UK

**Keywords:** airway allergy, cytokines, eosinophils, helminth immunomodulators

## Abstract

Type 2-high asthma is a chronic inflammatory disease of the airways which is increasingly prevalent in countries where helminth parasite infections are rare, and characterized by T helper 2 (Th2)-dependent accumulation of eosinophils in the lungs. Regulatory cytokines such as TGF-β can restrain inflammatory reactions, dampen allergic Th2 responses, and control eosinophil activation. The murine helminth parasite *Heligmosomoides polygyrus* releases a TGF-β mimic (*Hp*-TGM) that replicates the biological and functional properties of TGF-β despite bearing no structural similarity to the mammalian protein. Here, we investigated if *Hp*-TGM could alleviate allergic airway inflammation in mice exposed to *Alternaria alternata* allergen, house dust mite (HDM) extract or alum-adjuvanted ovalbumin protein (OVA). Intranasal administration of *Hp*-TGM during *Alternaria* exposure sharply reduced airway and lung tissue eosinophilia along with bronchoalveolar lavage fluid IL-5 and lung IL-33 cytokine levels at 24 h. The protective effect of *Hp*-TGM on airway eosinophilia was also obtained in the longer T-cell mediated models of HDM or OVA sensitisation with significant inhibition of eotaxin-1, IL-4 and IL-13 responses depending on the model and timepoint. *Hp*-TGM was also protective when administered parenterally either when given at the time of allergic sensitisation or during airway allergen challenge. This project has taken the first steps in identifying the role of *Hp*-TGM in allergic asthma and highlighted its ability to control lung inflammation and allergic pathology. Future research will investigate the mode of action of *Hp*-TGM against airway allergic eosinophilia, and further explore its potential to be developed as a biotherapeutic in allergic asthma.

## Introduction

Type 2-high asthma is a chronic inflammatory disease of the airways leading to cough, wheeze, shortness of breath, and chest tightness. This form of asthma is induced by early-life encounters with environmental allergens, including house dust mite (HDM) and *Alternaria alternata*, which evoke T helper 2 (Th2) cell responses [1]. Exuberant production of IL-4, IL-5, and IL-13 leads to asthma features with the accumulation of type 2-associated cells, such as eosinophils, in lung tissue [2, 3–5]. Eosinophil infiltration into the lung plays a causal role in augmentation of broader inflammation and is associated with asthma disease severity [1, 6, 7]. Targeting eosinophils has shown promise in alleviating asthma in clinical trials with therapeutic antibodies blocking type 2 cytokines or their receptors [3, 4, 8–14].

Eosinophils are regulated by a suite of cells, cytokines, and chemokines. In response to inhaled allergens, alarmin cytokines such as IL-33 are released by necrotic airway epithelial cell, triggering type 2 dendritic cells, which migrate from the lung tissue to the draining lymph node to induce Th2 differentiation [15]. Type 2 innate lymphoid cells (ILC2s) also respond to IL-33, and together with Th2 cells produce interleukin (IL)-5, to induce eosinophil differentiation, activation and recruitment into inflamed lungs [16]. Contemporaneously, IL-4 and IL-13 production stimulates the generation of eotaxins (e.g., Eotaxin-1/CCL-11), the main chemoattractants for eosinophils produced by lung epithelial cells, fibroblasts and smooth muscle cells [4, 17].

IL-5 is considered to be the major cytokine in eosinophil biology, mobilizing eosinophils from the bone marrow during allergic inflammation, and driving their activation and proliferation in peripheral tissues [1, 13, 18]. Together with IL-3 and granulocyte/macrophage colony-stimulating factor (GM-CSF), IL-5 can prolong eosinophil survival and abrogate their apoptosis [19]. In addition, specific subsets of dendritic cells (type 1 dendritic cells) attract eosinophils into the airways in asthma models, mediated by the chemokines CCL17 and CCL22 [20].

Another important cytokine in the biology of eosinophils, and more broadly in immune regulation, is TGF-β [21, 22]. TGF-β can help control the activation of eosinophils [23–25] and reduce their number in the airways [25, 26] by blocking the pro-survival effects of IL-3, IL-5 and GM-CSF and inducing their apoptosis [19, 27–29]. However, there have been contrasting reports on the effects of TGF-β in the inflamed airways; over-expression of TGF-β in mouse models protected against airway allergy [30], while conversely genetic ablation of TGF-β1 in CD11c+ cells resulted in enhanced allergic eosinophilia [20]. In a different model, however, loss of TGF-β1 expression in epithelial cells muted ILC2 activation and reduced allergic inflammation in mice [31]. Nevertheless, in most settings TGF-β is potently anti-inflammatory [21, 22], particularly through the induction of Foxp3^+^ T regulatory cells (Tregs), which are essential for induction of tolerance to allergens at mucosal surfaces [32–35] and are known to alleviate experimental airway allergy [36–39].

Type 2-high allergic asthma is increasingly prevalent in the more economically developed countries, particularly in urban communities, where helminth parasite infections are rare. Helminth parasites may broadly suppress type 2 responses to escape the arm of immunity that targets them for expulsion. Interestingly, children with helminth infections show reduced levels of allergic reactivity [40], which is linked to the expansion of Tregs [41] and their ability to suppress airway allergy in mouse models [36, 42]. Furthermore, treatment with anthelmintic drugs to clear parasites results in more rapid acquisition of allergic reactivity [43, 44]. We and others have screened helminth parasites for molecular mediators that might explain reduced allergic status; such products are likely to have been honed by evolution into effective biological inhibitors of the type 2 immune pathway that may offer novel treatments for human asthma [45, 46].

Focussing on *Heligmosomoides polygyrus*, the intestinal nematode with potent anti-allergic effects [41, 47, 48] we established that *H. polygyrus* excretory/secretory (HES) products suppress allergic airway inflammation, whether given at sensitisation or at challenge [49]. Two parasite proteins that block sensitisation have been identified as HpARI [50, 51] and HpBARI [52], both of which act on the IL-33 pathway. In searching for further anti-allergic parasite products, we considered a TGF-β mimic, *Hp*-TGM [53]. *Hp*-TGM shares no homology to any TGF-β family member, however it binds mammalian TGF-β receptors and induces Foxp3^+^ Tregs in vitro and in vivo [54–56]. The role of *Hp*-TGM in Type 2-high asthma remains unexplored.

In this study, we investigated the effects of *Hp*-TGM in murine models of allergic asthma elicited by *A. alternata* allergen, HDM or alum-adjuvanted Ovalbumin (OVA) protein. In the simplest model, intranasal administration of *Hp*-TGM in acute pulmonary inflammation following *A. alternata* exposure reduced pulmonary eosinophilia 24 h later. Moreover, in models of allergen challenge of sensitized mice, systemic administration of *Hp*-TGM potently reduced eosinophilic influx in the broncho-alveolar lavage fluid (BALF) and lung, both when given at the time of airway sensitization or at the time of airway challenge. Our data open the door to future research into therapeutic application of helminth-derived molecules to prevent the development or alleviate the symptoms of allergic asthma.

## Methods

### Reagents

Alternaria alternata extract (Greer Laboratories XPM1D3A25) was resuspended in PBS and concentration assessed by Pierce BCA assay (Thermo Fisher Scientific). HDM extract from *Dermatophagoides pteronyssinus* (Greer Laboratories XPB70D3A2.5) was resuspended in sterile PBS. Class IV Ovalbumin (Sigma, Gillingham, Dorset, UK) was lipopolysaccharide (LPS) depleted by Triton X-114 phase separation, and the levels of LPS present in HES and depleted OVA were below 0.1 and 0.01 IU LPS per mg protein, respectively by the Limulus Amoebocyte Lysate assay (Lonza, Slough, UK).

### Protein expression and purification

Construct encoding *Hp*-TGM was cloned into the pSec-TAG2A expression vector as previously described, with C-terminal myc and 6-His tags [53]. Purified plasmids were transfected into Expi293F™ cells, and supernatants collected 5 days later. Expi293F™ cells were maintained, and transfections carried out using the Expi293 Expression System according to manufacturer’s instructions (ThermoFisher Scientific). Expressed protein in supernatants were purified over a HisTrap excel column (GE Healthcare) and eluted in a gradient up to 500 mM imidazole. Eluted protein was then dialysed to PBS and filter-sterilized. The protein concentration was determined from A280 nM (Nanodrop, ThermoFisher Scientific), using a calculated extinction coefficient.

### Animals

BALB/cAnNCrl mice were purchased from Charles River, UK. RAG1^−/−^ mice and Foxp3-GFP mice were bred at the University of Glasgow. All mice were accommodated and procedures approved by the University of Glasgow Animal Welfare and Ethical Review Board, and performed under UK Home Office licences with institutional oversight performed by qualified veterinarians.

### Murine lung and BALF single cell suspension preparation

Lungs were flushed with four washes of 0.5 ml ice-cold PBS to collect BALF cells, followed by lung dissection for single cell preparation. Murine lungs were digested in 2 U/mL of Liberase TL (Roche, Burgess Hill, UK) and 80 U/mL DNase (Life technologies, Paisley, UK) at 37°C with agitation for 35 min. Digested tissue was passed through a 70 μm strainer and red blood cells lysed using Red Blood Cells Lysis Buffer (Sigma). Live cells were counted using a haemocytometer and dead cells excluded using trypan blue.

### Alternaria model

*Alternaria* allergen was used as a model of asthma as previously described [51, 57]. *Alternaria* allergen (10 μg) and *Hp*-TGM (5 μg) were administered intranasally to mice under isoflurane anaesthesia and tissues were harvested 24 h after initial *Alternaria* allergen administration.

### House dust mite model

HDM allergen was used as a model of asthma as previously described [58]. Mice were anaesthetized with iso-flurane, sensitized intranasally (i.n.) with 3 μg HDM in 50 μl PBS. After 7 days, mice were challenged with 10 μg HDM on five consecutive days under anaesthesia. Three days after the last challenge, mice were sacrificed, and tissues were harvested. *Hp*-TGM (1 μg) was administered i.n. or intraperitoneally (i.p.), as indicated.

### OVA/alum model

The OVA/Alum was used as a model of asthma as previously described [59]. Mice were anaesthetized with isoflurane and intraperitoneally administered 200 μl of PBS-Imject Alum containing 20 μg Ovalbumin (OVA) (final ratio of 1:1 Imject Alum to OVA). Day 7–9 post sensitisation, mice were briefly anaesthetised with isoflurane and intranasally (i.n.) administered 50 μl PBS containing 20 μg Ovalbumin. Twenty-four hours after the last challenge, mice were sacrificed and tissues were harvested. *Hp*-TGM (1 μg) was administered intraperitoneally (i.p.), as indicated.

### Flow cytometry staining

For surface staining, single cell suspensions from lung or BALF were washed in PBS and stained with Fixable Blue Live/Dead stain (Thermo Fisher). Cells were then blocked with anti-mouse CD16/32 antibody (Biolegend) and surface stained. BALF and lung cells were labelled with CD3 (FITC, clone 145-2C11, Biolegend), Siglec-F (PE, clone REA798, Miltenyi), Ly6G (PerCP-Cy5.5, clone 1A8, Biolegend), CD11c (AF647, clone N418, Biolegend), CD11b (Pacific Blue, clone M1/70, Biolegend), CD45 (AF700, clone 30-F11 Invitrogen), CD4 (PE-Dazzle, clone RM4.5, Biolegend), Lineage marker cocktail composed of FITC-labelled CD3 (clone 145-2C11, Biolegend), CD5 (clone 53–7.3, Biolegend), CD11b (M1/70.15, Invitrogen), CD19 (clone 6D5, Biolegend), CD49b (clone DX5, eBioscience) and GR1 (clone RB6-8C5, Biolegend), ICOS (PerCP Cy5.5, clone C398.4A, Biolegend), ST2 (APC, clone DIH9-2, Biolegend), CD25 (BV650, clone PC61, Biolegend), CCR3 (PerCP/Cyanine5.5, clone J073E5, Biolegend), and CD69 (PE, clone H1.2F3, Biolegend).

For intracellular cytokine staining, single lung cell suspensions were stimulated with PMA (500 ng/ml), ionomycin (1 μg/ml) and brefeldin A (10 μg/ml) for 4 h at 37° C, 5% CO2. Stimulated cells were surface stained, and permeabilised with IC permeabilization buffer following manufacturer instructions (eBioscience). For intracellular cytokine staining, cells were stained for IL-5 (PE, clone TRFK5, Biolegend) and IL-13 (PECy7, clone eBio13A, Biolegend). For Foxp3 (eF450, clone FJK-16s, eBioscience) staining, cells were fixed and permeabilised with Foxp3/transcription factor staining buffer following manufacturer instructions (eBioscience).

### RNA extraction, reverse transcription and qPCR

Lung tissue was placed in RNALater Stabilizing Solution (Thermo Fisher Scientific) and stored at −20°C. Lung tissues were then transferred from RNALater to TRIzol (Thermo Fisher Scientific) and homogenized using 3 mm stainless steel beads (Qiagen) in a TissueLyser II (Qiagen) at 25 Hz for 2 min.

Complementary DNA was made using qScript^®^ cDNA Synthesis Kit (Quantabio). Primers were pur-chased from Life Technologies and amplification reaction was carried out using PerfeCTa^®^ SYBR^®^ Green Super-Mix, Low ROX (Quantabio) in a 13 μl volume made up of 0.25 μl of nuclease free water, 0.25 μl forward primer, 0.25 μl reverse primer, 6.25 μl of 2x concentrated reaction mix and 6 μl DNA template (10 ng). Amplification was carried out using QuantStudio and 384-well plate (Applied Biosystems). PCR data were analysed using QuantStudio Real-Time PCR software v1.1 and the 2-ΔΔCT method. In brief, relative gene expression between different samples was calculated using the threshold cycles (CTs) generated by QuantStudio 384-well plate machine and software. ΔCT for each sample was calculated subtracting CT of the housekeeping gene (HPRT-1) from the CT of the gene of interest. To obtain the relative gene expression between control group and treatment, ΔΔCTs were then obtained subtracting the average of the control group ΔCTs (e.g., PBS) from the ΔCT of the sample. Subsequently, 2-ΔΔCT was calculated and plotted in a graph.

### Cytokine measurement

Cytokine measurements by Luminex assays (ProcartaPlex, ThermoFisher Scientific) and ELISAs for IL-4, IL-5, IL-13 (Ready-SET-go, ThermoFisher Scientific) and CCL-11 (Eotaxin-1; DuoSet, Bio-Techne) were carried out to manufacturer’s instructions.

### Statistical analysis

All data were analysed using Prism (Graphpad Software Inc.), one-way ANOVA with Tukey’s multiple comparisons post-test was used to compare multiple independent groups. ****p < 0.0001; ***p < 0.001; **p < 0.01; *p < 0.05; ns = not significant (*p* > 0.05). Where significant effects were observed for physiological outcomes (e.g., eosinophilia) similar group sizes were deemed sufficient to test individual parameters, such as cytokines.

## Results

### *Hp*-TGM inhibits innate allergic airway responses

**Figure 1:**
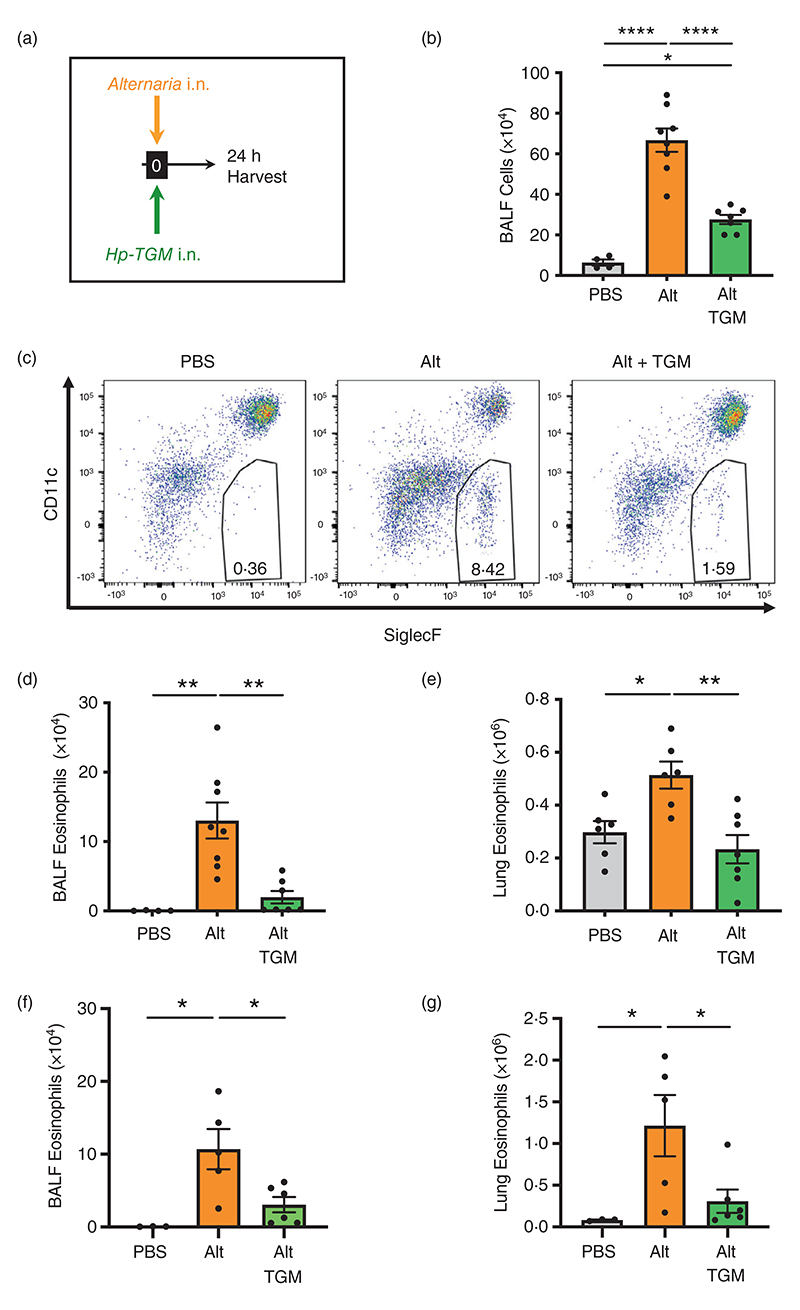
*Hp*-TGM reduces airway eosinophilia in response to *Alternaria* allergen. *Alternaria* allergen (10 μg) mixed with or without *Hp*-TGM (5 μg) was intranasally administered and mice culled 24 h later for recovery of bronchoalveolar lavage fluids (BALFs) and lung tissues. (a) Schematic representation of the experimental protocol. (b) Total cell numbers in BALF from control mice and those receiving *Alternaria* allergen (Alt) or *Alternaria* with *Hp*-TGM (Alt-TGM). (c) Representative flow cytometry plots of SiglecF vs. CD11c expression on CD45^+^ BALF cells from individual mice from the three experimental groups; percentage of eosinophils shown in inset boxes for each plot. (d) Numbers of eosinophils (CD45^+^CD11b^+^CD11c^−^Ly6C^−^Ly6G^−^SiglecF^+^) in BALF determined by flow cytometry. (d) Numbers of eosinophils (CD45^+^CD11b^+^CD11c^−^Ly6C^−^Ly6G^−^SiglecF^+^) in lung homogenates determined by flow cvtometrv. (f and g) Numbers of eosinophils (CD45^+^CD11b^+^CD11c^−^Ly6C^−^Ly6G^−^SiglecF^+^) in BALF (f) and lung homogenates (g) of RAG-1 deficient mice determined by flow cytometry. Results are pooled from two independent experiments each with 2–4 mice per group. All data were analvsed using Prism (Graphpad Software Inc.), one-wav ANOVA with Tukev’s multiple comparisons post-test was used to compare multiple independent groups. Mean values and SEM are indicated. ****p < 0.0001; **p < 0.01; *p < 0.05; non-significant differences not shown.

We first evaluated the ability of *Hp*-TGM to influence the early innate pro-allergic response to the fungal allergen *A. alternata* 24 h after intranasal administration. This model was previously shown to be highly sensitive to inhibition by total *H. polygyrus* HES, and key IL-33-pathway inhibitors in HES, *HpARI* and *HpBARI* [51, 52, 57]. In this system, we tested the effect of co-administration of *Hp*-TGM and *A. alternata* extract by the intranasal route (Figure 1a). We analysed differential cell responses by flow cytometry using the gating strategies shown for BALF (Supplementary Figure 1) and lung (Supplementary Figure 2) cells. BALF contents broadly represent responses of airway epithelial cells and resident or recruited immune cells to allergen exposure, while analyses of whole lung homogenates also reflect changes in the lung parenchyma.

In the BALF, *A. alternata* induced strong airway inflammation and eosinophilia which was highly suppressed by co-administration of *Hp*-TGM, as measured by total cellular infiltrate into the BALF (Figure 1b) and total eosinophils within the infiltrating population (Figure 1c,d). In the lung, eosinophilia developed within 24 h of *A. alternata* treatment, but this was abolished by co-administration of *Hp*-TGM (Figure 1e). *Hp*-TGM was also effective in abrogating *A. alternata*-induced eosinophilia in RAG-1-deficient mice (Figure 1f,g), confirming that innate responses were suppressed directly without involvement of adaptive lymphocytes. Furthermore, *Hp*-TGM inhibition of *A. alternata*-induced pulmonary eosinophilia was observed to act in a dose-dependent manner with diminution evident even when 5 ng of the parasite mediator was administered (Supplementary Figure 3A).

**Figure 2:**
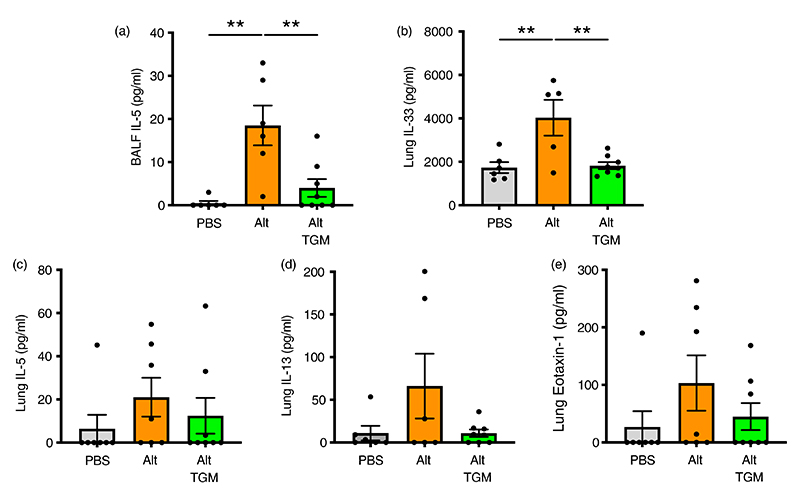
Cytokine responses in Alternaria-challenged airways. Mice were treated with *Alternaria* allergen with or without *Hp*-TGM as described for Figure 1 and samples recovered at 24 h. (a) IL-5 cytokine measured in cell-free BALF supernatants. (b–e) IL-33 (b), IL-5 (c), IL-13 (d), eotaxin-1 (e) cytokines measured in lung homogenates. Results are pooled from two independent experiments each with 3–4 mice per group. All data were analysed using Prism (Graphpad Software Inc.), one-way ANOVA with Tukey’s multiple comparisons post-test was used to compare multiple independent groups. Mean values and SEM are indicated. **p < 0.01; *p < 0.05; non-significant differences not shown.

We then measured type 2 cytokines in BALF and lung homogenate; in BALF, IL-5 levels induced by *A. alternata* were substantially reduced when the allergen was coadministered with *Hp*-TGM (Figure 2a). BALF IL-13 levels were marginally above the limit of detection in some, but not all animals (data not shown), and *Hp*-TGM exerted no significant effect. In the lung, IL-33 in lung homogenates was fully suppressed to baseline levels in the presence of *Hp*-TGM (Figure 2b), while IL-5, IL-13 and eotaxin-1 were reduced to different degrees, albeit falling short of significance (Figure 2c–e). We also analysed type 2 innate lymphoid cells (ILC2s), which are known to respond to IL-33 and contribute IL-13 and IL-5 to the early allergic response, but noted no overt effect of *Hp*-TGM on their expression by flow cytometry (Supplementary Figure 3B,C).

### *Hp*-TGM is protective in a T cell-dependent model of airway allergy

**Figure 3:**
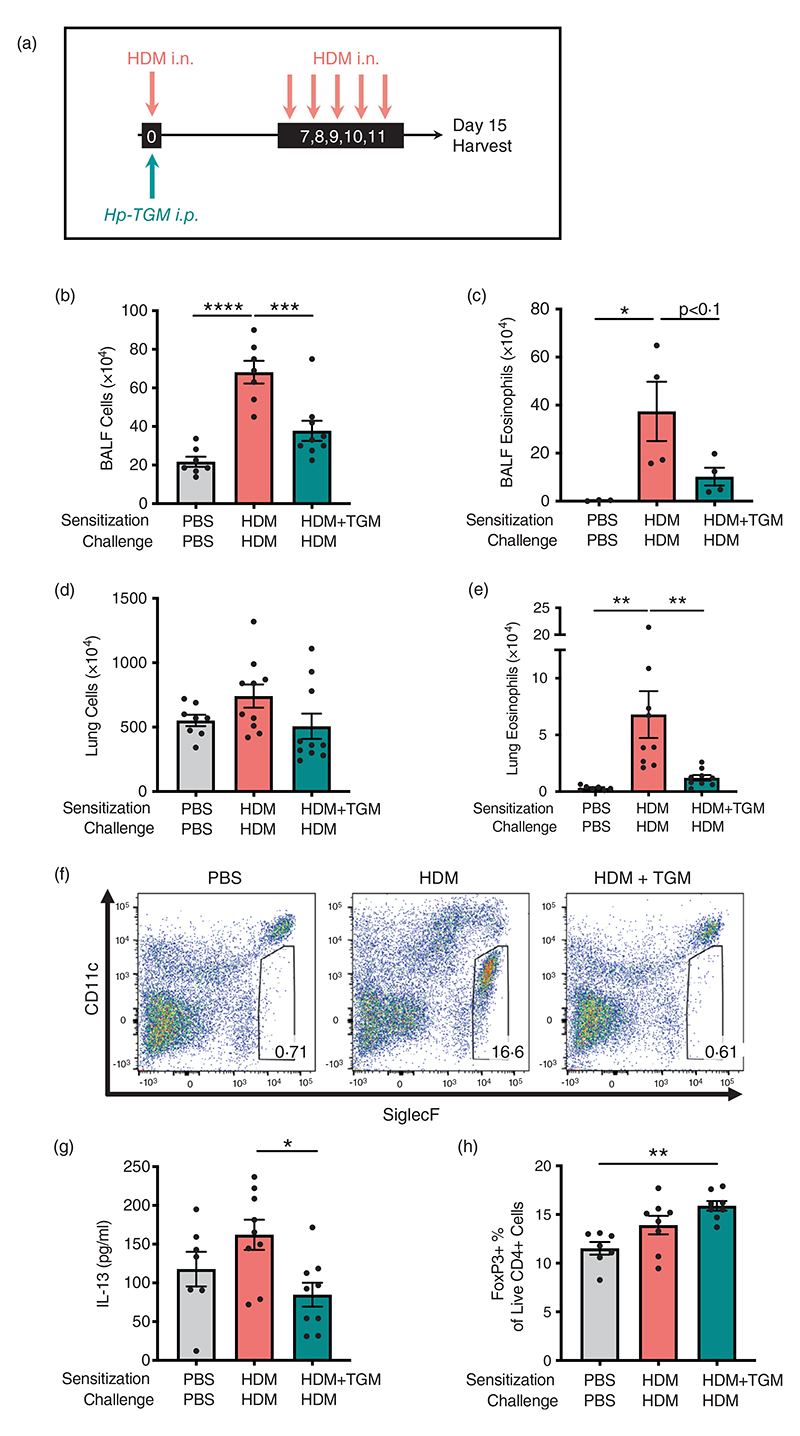
*Hp*-TGM reduces allergic sensitization to house dust mite. Mice were sensitized with house dust mite allergen (3 μg) intranasally (i.n.) and *Hp*-TGM (1 μg) was administered intraperitoneally (i.p.) at day 0. Allergic responses to HDM were restimulated daily from day 7 to day 11 (10 μg) and mice culled at day 15. (a) Schematic representation of the experimental protocol. (b and c) Total cell (b) and eosinophil (c) cell numbers were enumerated in the BALF. (d) Total cell numbers enumerated in lung homogenates. (e and f) Eosinophil cell numbers enumerated in lung homogenates (e) and representative flow cytometry plot of SiglecF vs. CD11c expression on lung cells gated on live CD45^+^ cells. Percentage of eosinophils shown in inset boxes for each plot (f). (g) IL-13 measured by ELISA in lung homogenates. (h) Foxp3 expression on lung cells gated on live CD4^+^ cells from control mice and those receiving HDM with or without *Hp*-TGM. Data are from ≥2 independent experiments (*n* = 6–9), except panel (c) which is from one representative experiment (*n* = 4–5). All data were analysed using Prism (Graphpad Software Inc.), one-way ANOVA with Tukey’s multiple comparisons post-test was used to compare multiple independent groups. Mean values and SEM are indicated. ****p < 0.0001; ***p < 0.001; **p < 0.01; *p < 0.05; nonsignificant differences not shown.

Because allergy is primarily mediated by a type 2 adaptive immune response, we then tested *Hp*-TGM in a T cell-dependent model of airway sensitization and challenge, using HDM extract [60], with intraperitoneal administration of *Hp*-TGM during allergen sensitization (day 0) or at each day of challenge (day 7–11) (Figure 3a). In this context, a single exposure to *Hp*-TGM on the day of sensitisation was sufficient to strongly inhibit total cellular influx into the BALF when assessed 4 days after the final challenge dose (Figure 3b). A substantial reduction in eosinophilia was seen although not reaching statistical significance (Figure 3c). In the lung, while total cellularity did not differ greatly (Figure 3d), eosinophilia (Figure 3e,f) was strongly down-modulated in animals receiving *Hp*-TGM at the time of sensitization.

Levels of type 2 cytokines in lung homogenates on day 15 were then analysed. *Hp*-TGM administration strongly inhibited IL-13 responses in animals receiving HDM (Figure 3g), while other cytokines (IL-4, IL-5 and eotaxin-1) were not significantly induced by HDM in these experiments and no significant differences in these cytokines were observed with *Hp*-TGM administration (data not shown). As *Hp*-TGM is a potent inducer of CD25^+^Foxp3^+^ regulatory T cells, we also evaluated their levels in the lungs of mice in these experiments. As shown in Figure 3h, the proportion of Foxp3^+^ Tregs differed only slightly between the groups, although compared to naïve controls there was a significant increase in mice receiving *Hp*-TGM on the day of allergic sensitization with HDM.

### *Hp*-TGM sharply reduces allergic eosinophilia in response to allergen challenge

**Figure 4:**
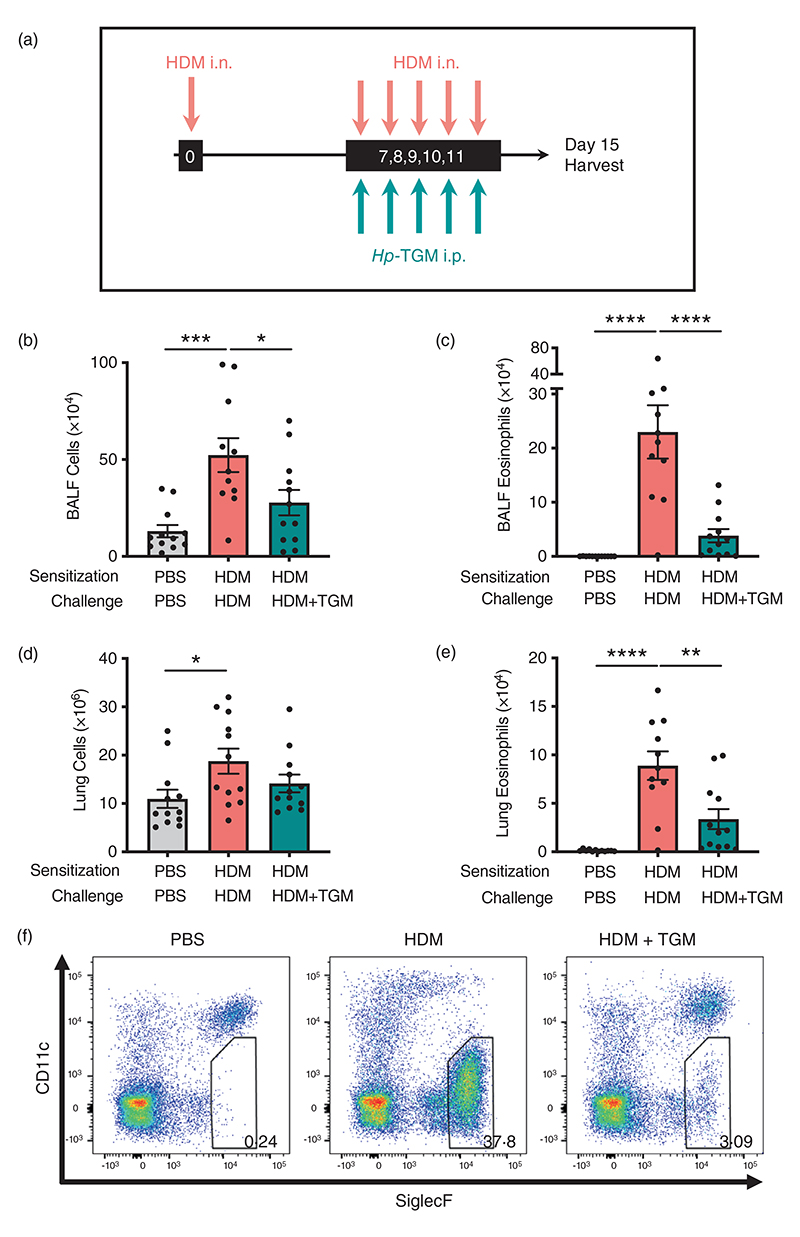
*Hp*-TGM administrated at challenge reduces allergic responses to house dust mite. Mice were sensitized with house dust mite allergen (3 μg) intranasally at day 0. Allergic responses to HDM were recalled daily from day 7 to day 11 (10 μg) and *Hp*-TGM (1 μg) was administered intraperitoneally every day of challenge. Mice were then culled at day 15. (a) Schematic representation of the experimental protocol. (b and c) Total cell (b) and eosinophil (c) numbers were enumerated in BALF. (d) Total cell numbers enumerated in lung homogenates. (e and f) Eosinophil cell numbers enumerated in lung homogenates (e) and representative flow cytometry plot of SiglecF versus CD11c expression on lung cells gated on live CD45^+^ cells. Percentage of eosinophils shown in inset boxes for each plot (f). Data are from ≥2 independent experiments (*n* = 7-9). All data were analysed using Prism (Graphpad Software Inc.), one-way ANOVA with Tukey’s multiple comparisons post-test was used to compare multiple independent groups. Mean values and SEM are indicated. ****p < 0.0001; ***p < 0.001; **p < 0.01; *p < 0.05; nonsignificant differences not shown.

To further investigate the potential of *Hp*-TGM as a therapeutic option for airway allergy, we next tested its ability to dampen allergic inflammation in previously sensitized animals. Mice were sensitized with HDM at day 0 and challenged with the same allergen for five consecutive days from day 7 to day 11 after sensitization. On the same day of each HDM challenge, mice received either *Hp*-TGM or PBS i.p. (Figure 4a). Four days following the final challenge, total cell influx, lymphocyte infiltration and eosinophilia were measured in the BALF and lung. In mice that received *Hp*-TGM, total cell numbers were sharply reduced in the BALF (Figure 4b) with a highly suppressive effect on eosinophilia (Figure 4c). In the lung, although the total cellularity was not greatly reduced (Figure 4d), a strong inhibition of eosinophilia was again evident (Figure 4e,f).

Analysis of BAL and lung neutrophil (Supplementary Figure 4A,C, respectively) and alveolar macrophage (Supplementary Figure 4B,D, respectively) populations showed similar responses in mice challenged with HDM, irrespective of the presence or absence of *Hp*-TGM administration. Similarly, key cell types in the pulmonary tissues found only modest differences in Th2 (Supplementary Figure 5I) and ILC2 (Supplementary Figure 5J) cell numbers, neither of which reached statistical significance.

### Suppression of eosinophilia by systemic *Hp*-TGM is associated with significant reduction of eotaxin-1 and IL-4 responses, rather than induction of regulatory responses

**Figure 5:**
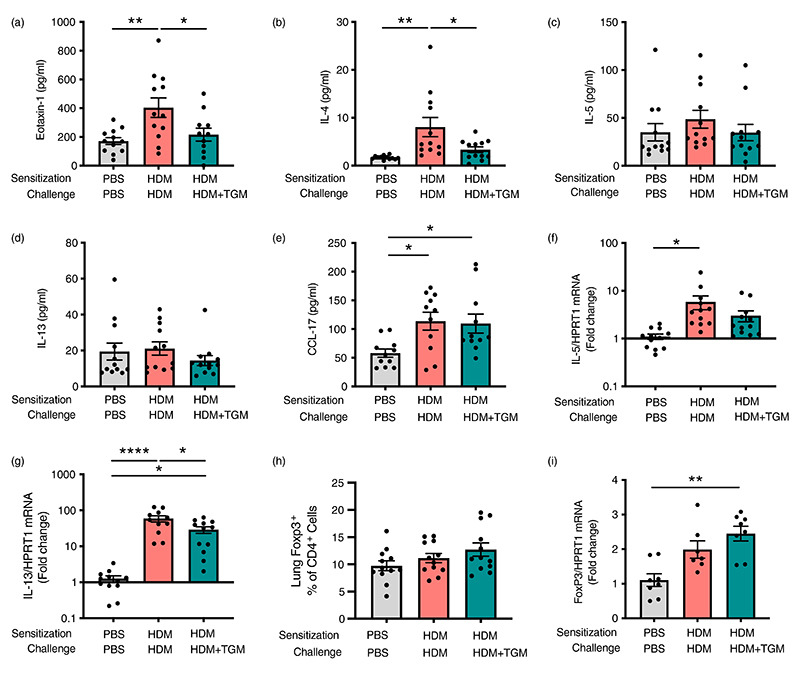
Inhibition of eosinophilia by *Hp*-TGM is associated with lower IL-4 and eotaxin-1 responses. Using the same protocol as previously described (Figure 4a), type 2 cytokine and regulatory gene responses were assessed by Luminex and/or qPCR. (a–e) Lung homogenate levels of eotaxin-1, IL-4, IL-5, IL-13 and CCL17 were measured. (f and g) Expression of *Il5 and Il13* transcripts. (h) Percentage of Foxp3+ within all CD4+ T cells. (i) Expression of *Foxp3* transcripts in lung homogenates. Data are from ≥2 independent experiments, Data shown are pooled from two experiments (*n* = 7–9). All data were analysed using Prism (Graphpad Software Inc.), one-way ANOVA with Tukey’s multiple comparisons post-test was used to compare multiple independent groups. Mean values and SEM are indicated. ****p < 0.0001; **p < 0.01; *p < 0.05; non-significant differences not shown.

We then examined if the effect of *Hp*-TGM during allergen challenge was accompanied by a modulation of type 2 cytokines in the lung tissues. Most notably, eotaxin-1 (Figure 5a) and IL-4 (Figure 5b) were significantly attenuated in *Hp*-TGM-treated mice, although at this time point IL-5 (Figure 5c) and IL-13 (Figure 5d) were not different between groups. An increase in CCL17 chemokine levels in HDM-allergic mice was similar irrespective of *Hp*-TGM administration (Figure 5e). We also examined mRNA transcript levels for these and other cytokines, finding no effect on eotaxin-1, IL-4 (Supplementary Figure 5A,B), or IL-5 (Figure 5f), but a significant decrease in IL-13 transcripts following *Hp*-TGM treatment (Figure 5g). Of note, no differences in BALF cytokines were observed (data not shown). We also measured GM-CSF and IL-3 protein levels in lung homogenates, however, no effect of *Hp*-TGM were observed on these molecules (Supplementary Figure 5C,D).

In addition, we investigated if *Hp*-TGM effect on allergic eosinophilia involved induction of Tregs and their regulatory cytokines, since we and others have previously shown their importance in the suppression of airway allergy [36, 61, 62]. However, the percentage of Tregs in CD4^+^ T cells (Figure 5h) and levels of Foxp3 mRNA (Figure 5i) were only marginally increased following parenteral *Hp*-TGM and did not reach significance. Similarly, IL-10 protein and mRNA transcript levels (Supplementary Figure 5E,F), and TGFβ mRNA levels (Supplementary Figure 5G) were unchanged by *Hp*-TGM administration. Interestingly, HDM treatment resulted in a significant reduction in IL-4Rα transcripts compared to both control and *Hp*-TGM-treated mice (Supplementary Figure 5H), which may represent receptor downregulation following a higher level of activation.

### *Hp*-TGM also reduces airway eosinophilia in the ovalbumin allergy model

**Figure 6:**
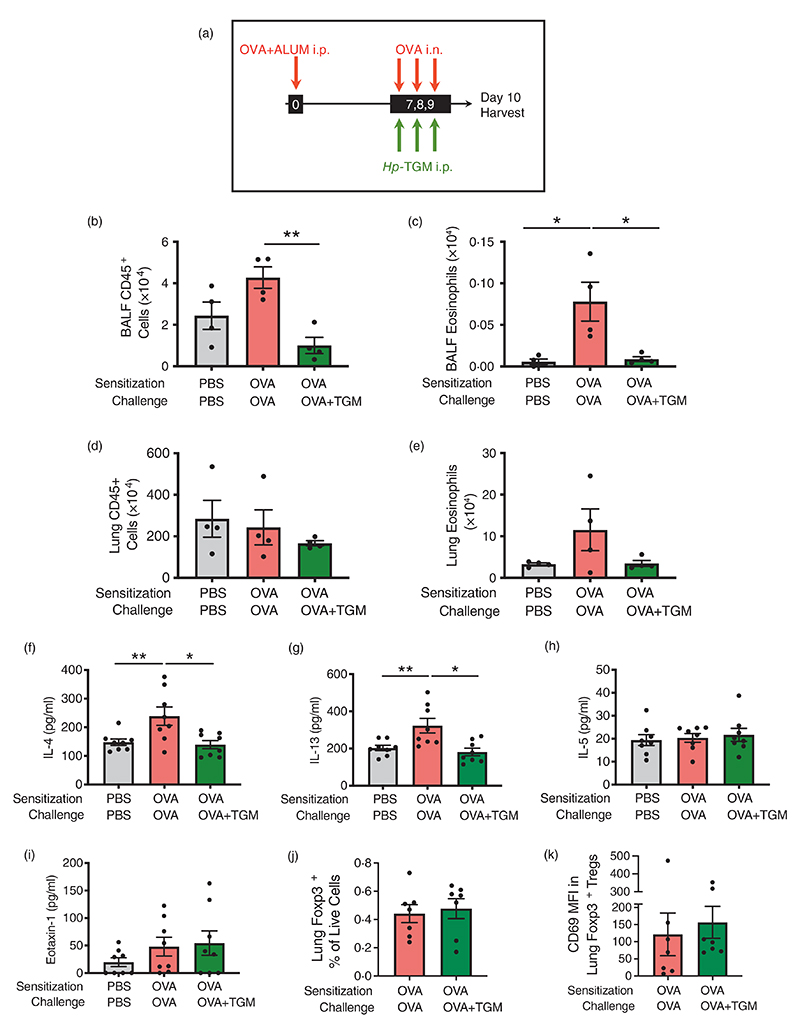
*Hp*-TGM blocks allergic eosinophilia during an OVA challenge. Mice were sensitized with Ovalbumin (OVA, 20 μg) intraperitoneally at day 0. OVA was then intranasally administered daily from day 7 to day 9 (20 μg) and *Hp*-TGM (1 μg) was administered intraperitoneally every day of challenge. Mice were then culled at day 10. (a) Schematic representation of the experimental protocol. (b–e) CD45^+^ cell and eosinophil cell numbers were enumerated in BALF (b and c) and lung (d and e). (f–i) Type 2 cytokines were measured in lung homogenates, showing IL-4, IL-13, IL-5 and eotaxin-1. (j) Frequency of Foxp3^+^ Tregs in lung homogenates of mice sensitized and challenged with OVA, in the absence or presence of *Hp*-TGM administration. (k) Expression of CD69 within the Foxp3^+^ Treg population in lung homogenates of mice sensitized and challenged with OVA, in the absence or presence of *Hp*-TGM administration. Data are from ≥2 independent experiments. Data shown are pooled from two experiments (*n* = 7–9). All data were analysed using Prism (Graphpad Software Inc.), one-way ANOVA with Tukey’s multiple comparisons post-test was used to compare multiple independent groups. Mean values and SEM are indicated. **p < 0.01; *p < 0.05; non-significant differences not shown.

Although ovalbumin-induced models are less clinically-relevant than airway allergen models, since sensitization is achieved by intraperitoneal administration of OVA protein emulsified in alum, they are characterized by a Th2 response with IL-4, IL-5, and IL-13 production, and are independent of epithelial-derived alarmin cytokines, such as IL-33 [1]. To check if parenteral *Hp*-TGM could also control eosinophilia in this allergy model, we administered a pre-mixed OVA/Alum solution intraperitoneally to mice at day 0, and recalled responses to OVA by administering it intranasally from day 7 to day 9 following injections with *Hp*-TGM 3 h earlier on the same day. On day 10, mice were culled, and airway tissues harvested (Figure 6a). In this setting, *Hp*-TGM significantly decreased CD45^+^ cell influx in the BALF (Figure 6b) and almost completely abolished eosinophilia (Figure 6c). In the lung, cellularity was little changed (Figure 6d) and while there was a strong trend for reduction of eosinophilia in the presence of *Hp*-TGM, this did not reach significance (Figure 6e). In addition, IL-4 and IL-13 were significantly reduced in lung homogenates of mice receiving *Hp*-TGM, while IL-5 and eotaxin-1 responses were little affected (Figure 6f–i). No significant differences in CCR3 (eotaxin-1 receptor) expression were seen between the different groups (Supplementary Figure 6A,B). Furthermore, no significant changes were seen in the profile of lung Foxp3^+^ Tregs as a results of *Hp*-TGM treatment, either in terms of frequency (Figure 6j) or induction of the activation marker CD69 within the Treg population (Figure 6k). Taken together, these data support the hypothesis of a regulation of T cell-mediated allergic airway eosinophilia by *Hp*-TGM via modulation of IL-4 and IL-13 responses.

## Discussion

Helminth parasites are frequently associated with protection from allergic reactivity [40, 42, 63, 64]. Although deliberate infection with helminths has been trialled for the alleviation of inflammatory symptoms [65–67], future helminth-derived therapies will require defined products which can be individually evaluated as potential pharmacological agents [45]. In this report, we show that the helminth-derived TGF-β mimic, *Hp*-TGM, potently suppressed allergic eosinophilia in three different experimental models of airway inflammation, in each case revealing specific cellular and cytokine modulation occurring in vivo.

In the first model, we appraised short-term innate responses 24 h following *A. alternata* exposure. Eosinophilia was sharply reduced by *Hp*-TGM co-administration, indicating direct effect(s) on innate cell populations, together with overall diminution of type 2 cytokines, in particular BALF IL-5 and lung IL-33, the latter known to be an essential driver of immune responses in this model [68–70]. In mice, IL-33 is predominantly released by stromal cells in the lung tissue, including epithelial cells, fibroblasts, and endothelial cells [71, 72], while in mast cells and dendritic cells IL-33 can be induced under inflammatory conditions [73, 74]. Ongoing studies aim to determine which of these populations may be down-regulated by *Hp*-TGM in this mode. Lung ILC2s release IL-5 and IL-13 in response to IL-33 [75, 76], but were not significantly affected by *Hp*-TGM. One cell type that also produces IL-5 and IL-13 in response to IL-33 is the mast cell [77], and as TGF-β has been shown to suppress IL-33–induced mast cell function in the context of asthma [78], this population is a prime candidate for further investigation.

We next tested *Hp*-TGM effects in airway allergy models where type 2 adaptive immune responses are involved and found significant protection when *Hp*-TGM was administered systemically. Suppression of eosinophilia was marked, both when *Hp*-TGM was given at the time of sensitization, or during repeated challenge, and again we found significant inhibition of eotaxin-1, IL-4 and/or IL-13 responses, depending on the model and the time-points examined. Similar effects on eosinophils were observed with HDM, or the model antigen Ovalbumin (OVA). However, in none of the protocols did *Hp*-TGM particularly affect Th2 and ILC2 cell numbers, and its effects on Foxp3^+^ regulatory T (Treg) cells were modest at best. This is in accordance with previous data from our group showing that administration of HES in allergic asthma models suppressed airway eosinophilia without inducing Treg responses [49].

Eotaxin-1 and IL-4 are important cytokine in eosinophil migration to inflamed sites. Indeed, IL-4 has been shown to induce the expression of adhesion molecules ICAM-1 (intercellular cell adhesion molecule-1) and VCAM-1 (vascular cell adhesion molecule-1) that allow eosinophils to exit the inflamed airways [79, 80], as well as to promote eosinophil chemotaxis by inducing eotaxin-1 in epithelial cells. We previously reported reduction of eotaxin-1 in allergy models in the presence of HES [49], which we now suggest may be mediated by *Hp*-TGM.

The source of IL-4 cytokine targeted by *Hp*-TGM in our models remain unknown, however, T follicular helper cells (Tfh) may well be the primary target of *Hp*-TGM since they are the main producer of IL-4 in allergic asthma [70, 81, 82]. We have yet to investigate the possible involvement of other immune cells known to produce IL-4 in the context of asthma, such as basophils [83–87] or activated ILC2s [70]. Notably, we found that *Hp*-TGM not only reduced lung IL-4 cytokine levels, but also downregulated IL-4Rα gene expression to baseline. Within the T cell population, which drives the response to HDM, IL-4Rα downregulation is induced by TCR engagement which renders the cell unresponsive to IL-4 [88]. Our data therefore suggest that *Hp*-TGM may limit Th cell antigenic stimulation, as part of a wider picture in which administration of HES in allergy models reduces Th2 responses without skewing responses towards Th1 cells [49].

Finally, in all models tested, *Hp*-TGM could have affected the eosinophil population directly. Indeed, TGF-β has been shown to downregulate eosinophil responses by various means, for example, by inducing their apoptosis [22, 27, 28], limiting their differentiation from bone marrow precursors, limiting their proliferation by antagonizing the effect of IL-3, IL-5 and GM-CSF [19, 22, 89, 90], by limiting their activation via downregulation of the eosinophils activation marker HLA-DR [91], or by retaining them in noninflamed tissues by enhancing expression of the chemokine receptor CXCR4 [22, 92]. Although we did not directly compare the effect of *Hp*-TGM to TGF-β, we have previously compared the effect of TGF-β and HES in similar asthma models [49, 93]. While intravenous recombinant TGF-β was shown to suppress development of Th2-mediated pathology, including airway eosinophilia the effect was mediated by the induction of Tregs [93]. Intranasal TGF-β did not show any suppression of eosinophilic BALF nor modulated Th2 cytokines [49]. In our models, systemic *Hp*-TGM did not significantly expand Treg frequencies nor modulated IL-3, IL-5 or GM-CSF responses. Thus, it is possible that *Hp*-TGM could have a different mode of action than TGF-β to control airway eosinophilia.

In summary, we present data showing that *Hp*-TGM administration can recapitulate generalized anti-allergic effects that were previously found for the complex mixture of *H. polygyrus* ES products (HES) [49, 93]. Airway epithelial cells are likely to be central in *Hp*-TGM effect on airway eosinophilia since *Hp*-TGM reduced the levels of IL-33 cytokine in the *Alternaria* model and eotaxin-1 levels in the HDM model, both of which are primarily epithelial-derived and underpin eosinophil accumulation in the allergic lung [70]. To our knowledge this is the first time that a parasite-derived protein has been described to reduce airway allergic eosinophilia on allergen rechallenge by acting on epithelial-derived cytokines and IL-4 responses rather than on the classical IL-5 cytokine response. We believe that *Hp*-TGM may contain possible preventive or therapeutic potential for human allergic airway disease, as targeting eosinophil infiltration in the lung has already proven to be successful in controlling type 2-high asthma in clinical trials [3, 4, 8, 9, 11–13]. Future work will explore this possibility in depth.

## Supplementary Material

Supplementary material

## Data Availability

The data that support the findings of this study are available from the corresponding author upon reasonable request.
