# Supplementary material for: Suppression of airway allergic eosinophilia by Hp-TGM, a helminth mimic of TGF-β
Source: Immunology. Author manuscript; Available in PMC 2023 Feb 16. (PMC9885513; doi:10.1111/imm.13528)

Supplementary Figure 1. Gating strategy for T cells, eosinophils, neutrophils and alveolar macrophages in BALF samples.

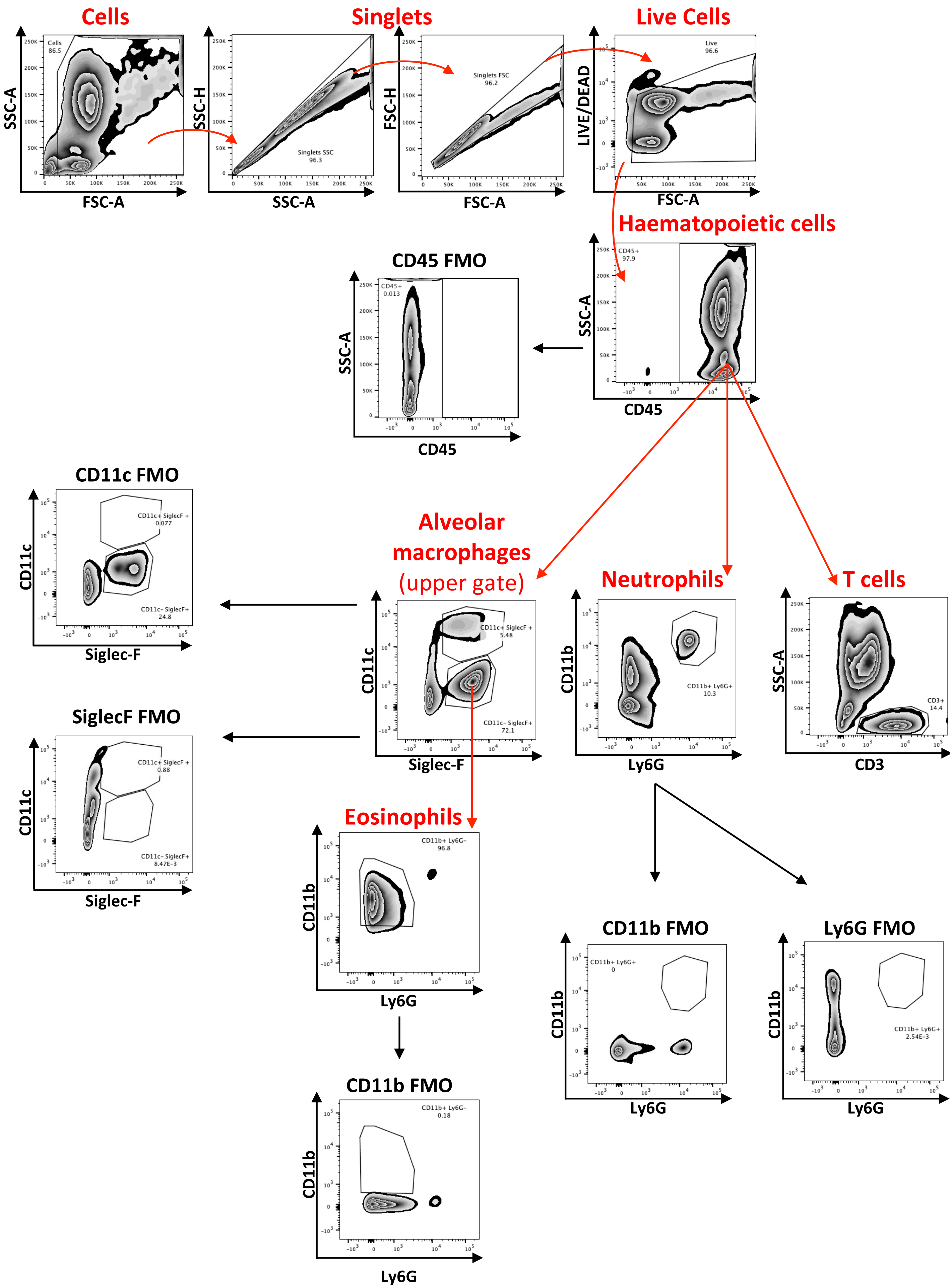

Supplementary Figure 2. Gating strategy for T cells, eosinophils, neutrophils and alveolar macrophages in lung samples.

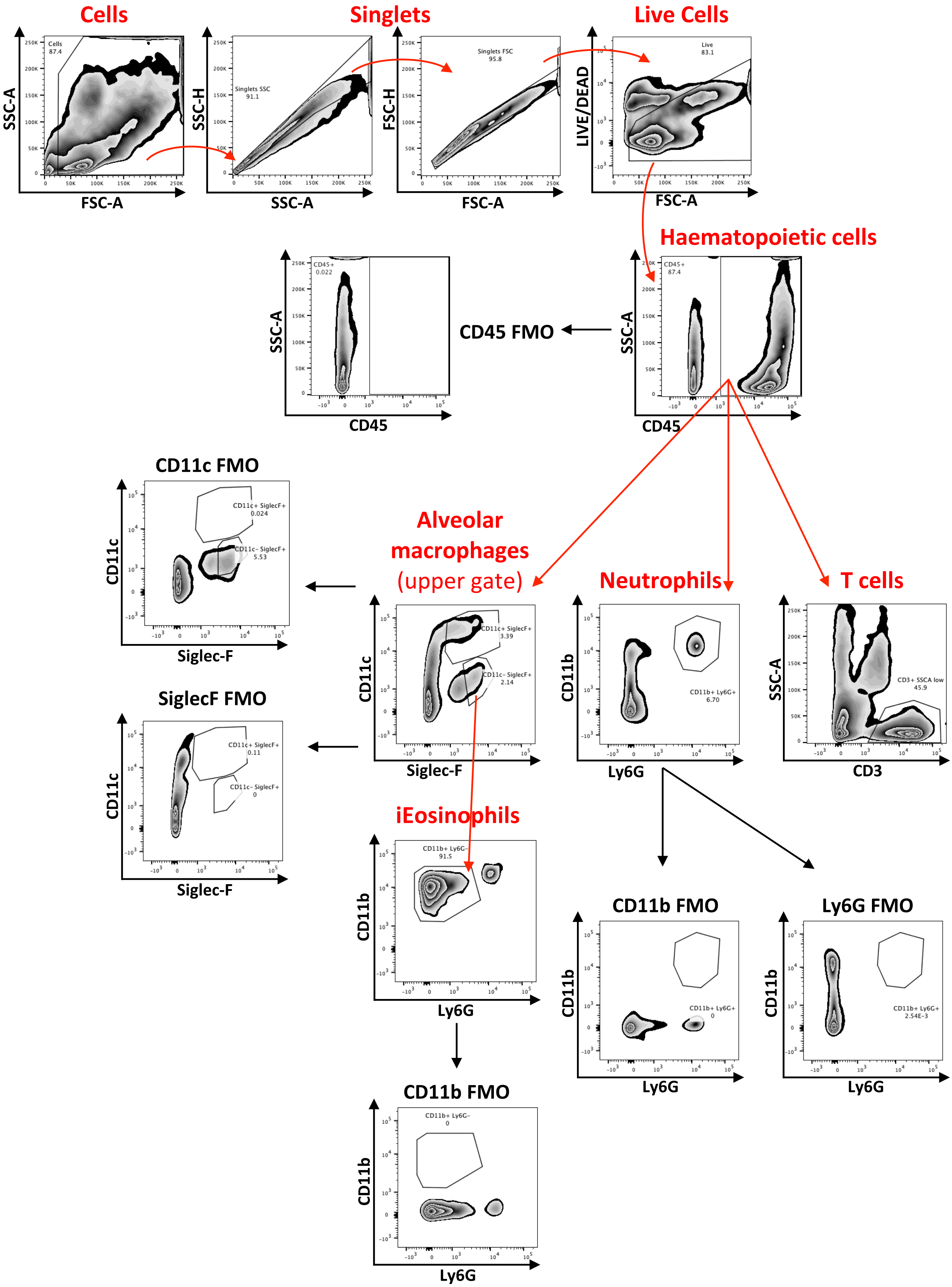

Chauché *et al.* Suppl Figure 3

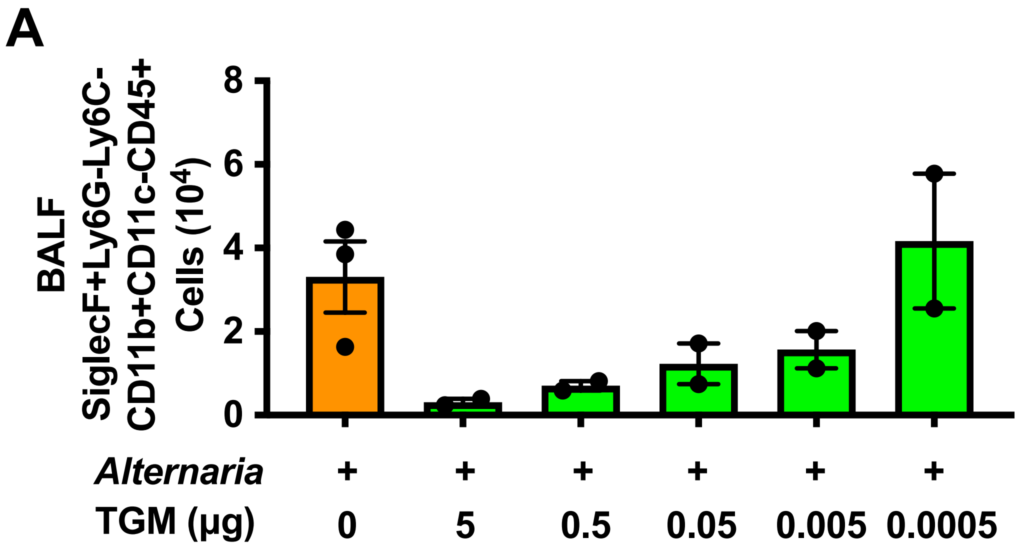

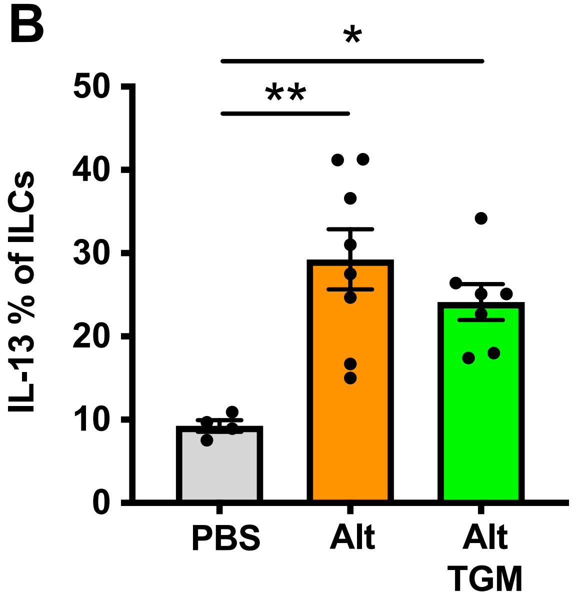

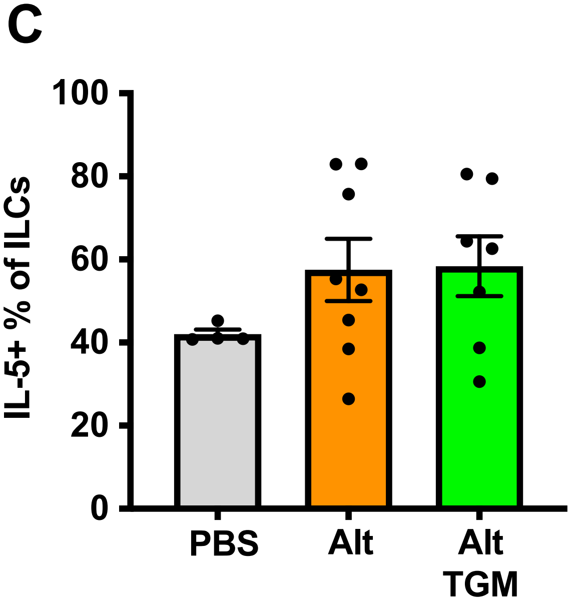

Chauché *et al.* Suppl. Figure 4

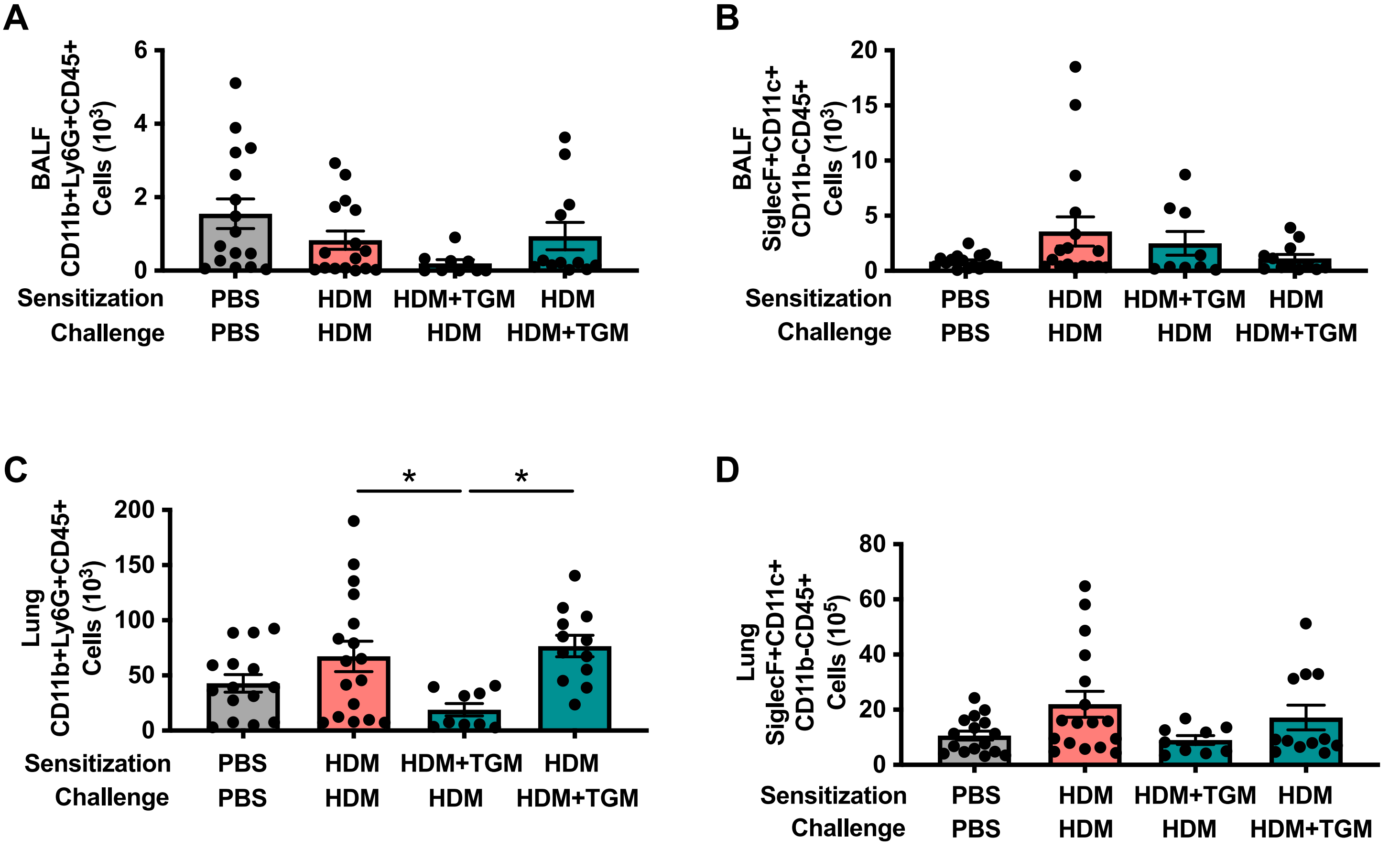

Chauché *et al.* Suppl. Figure 5

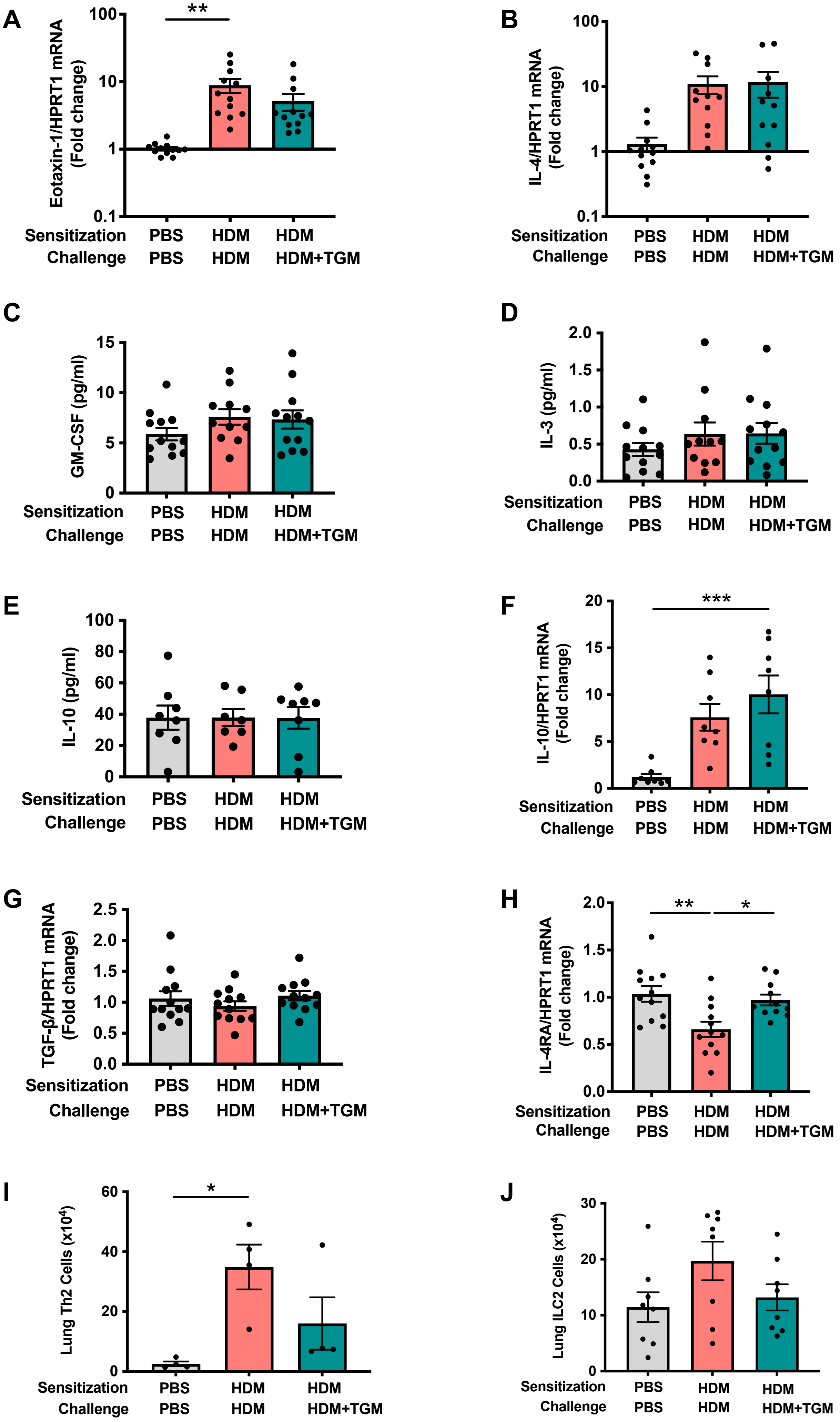

Chauché *et al.* Suppl. Figure 6

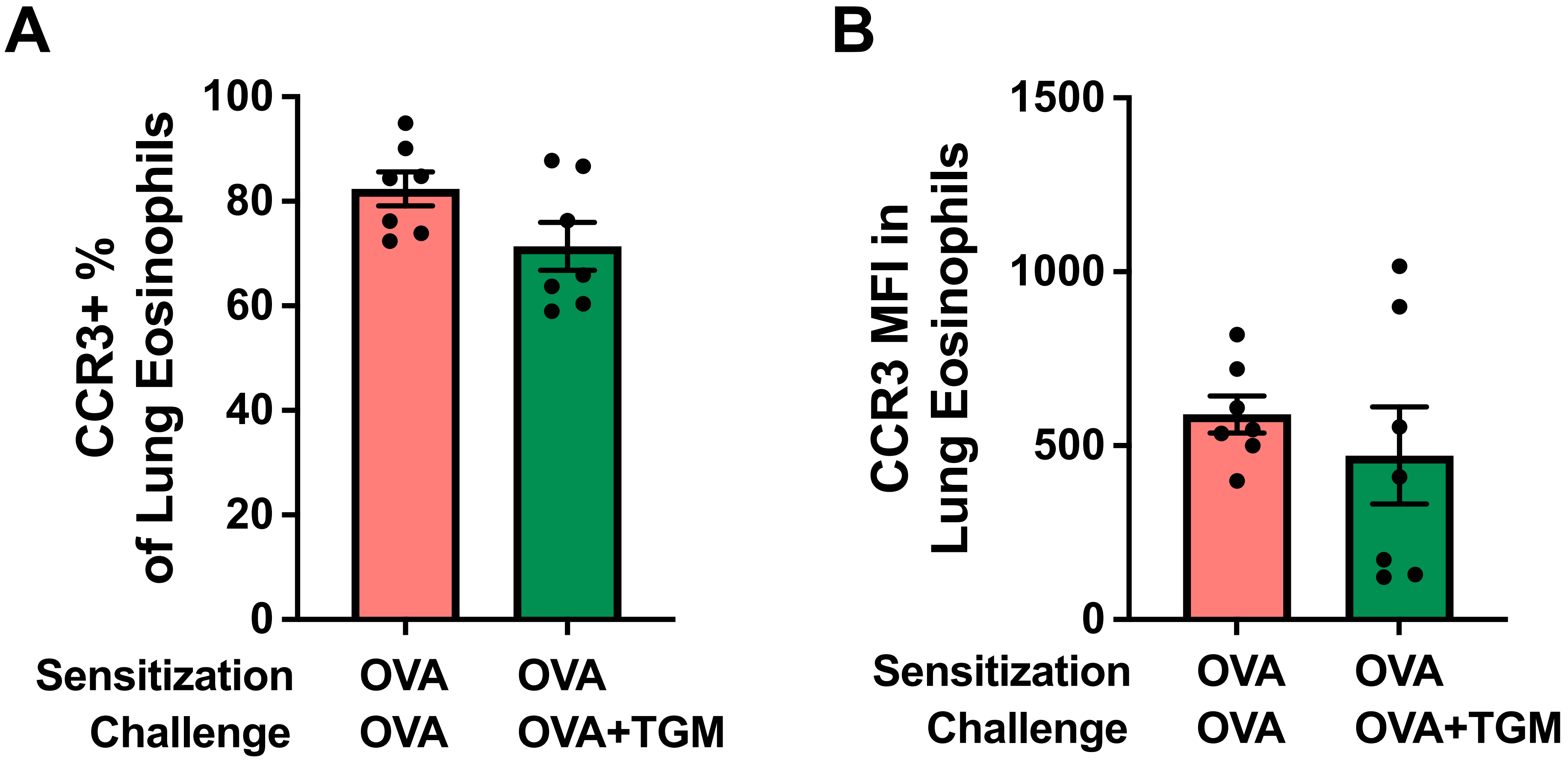

Supplement: Supplementary material [file EMS163680-supplement-Supplementary_material.pdf]
